# Supplementary material for: Validating Accuracy of an Internet-Based Application against USDA Computerized Nutrition Data System for Research on Essential Nutrients among Social-Ethnic Diets for the E-Health Era
Source: Nutrients. 2022 Jul 31;14(15):3168. doi: 10.3390/nu14153168 (PMC9370220; doi:10.3390/nu14153168)
Supplement: Supplementary file 1 [file nutrients-14-03168-s001.zip › nutrients-1805443-supplementary.pdf]

Supplementary Table S1: Bias and agreement between internet-based applications and Nutrition Data System for Research per domains of caloric ranges, energy nutrients, and various diets for major nutrients (*N*=131).

**Parameters (n)**	**Calories, kcal**<br>*r* (95%CI)<br>%diff M ± SD	**Carb, g**<br>*r* (95%CI)<br>%diff M ± SD	**Protein, g**<br>*r* (95%CI)<br>%diff M ± SD	**Fat, g**<br>*r* (95%CI)<br>%diff M ± SD	**Sat Fat, g**<br>*r* (95%CI)<br>%diff M ± SD	**Cholesterol, g**<br>*r* (95%CI)<br>%diff M ± SD	**Fiber, g**<br>*r* (95%CI)<br>%diff M ± SD
**Caloric Ranges**							
<1000 (63)	0.75** (0.62-0.84)<br>-4.26**± 10.01	0.85**(0.77-0.91)<br>-0.70** ± 12.55	0.80**(0.68-0.87)<br>-6.27** ± 11.35	0.75**(0.61-0.84)<br>-10.58**± 13.73	0.68**(0.53-0.79)<br>-14.81** ±15.63	0.95**(0.91-0.97)<br>-6.37** ± 16.24	0.89**(0.83-0.93)<br>8.23**± 19.49
1000-2000 (50)	0.76**(0.60-0.85)<br>-4.10**± 11.67	0.94**(0.89-0.96)<br>5.15**± 11.60	0.92**(0.86-0.95)<br>-3.03**± 9.04	0.56**(0.33-0.72)<br>-14.34**± 17.19)	0.68**(0.50-0.8)<br>-12.52** ± 16.04)	0.92**(0.86-0.95)<br>-2.08** ± 10.57	0.98**(0.97-0.99)<br>10.86** ± 10.64
>2000 (18)	0.57*(0.14-0.82)<br>-11.56 ± 25.71	0.55*(0.15-0.8)<br>-9.98 ± 29.51	0.65*(0.29-0.85)<br>-12.03* ± 23.09	0.73**(0.41-0.89)<br>-16.16**± 23.74	0.76**(0.48-0.9)<br>-13.62** ± 22.15	0.70**(0.37-0.87)<br>-7.27 ± 23.06	0.43(-0.02-0.73)<br>-10.21 ± 35.72
**Energy Nutrients**							
Fat	0.61**(0.43-0.74)<br>-12.37**± 15.54	0.56**(0.37-0.71)<br>-3.40 ± 20.26	0.70**(0.55-0.8)<br>-9.80** ±15.08	-	0.70**(0.56-0.81)<br>-24.48**± 14.86	0.74**(0.61-0.84)<br>-9.84** ± 16.43	0.60**(0.41-0.74)<br>3.39 ± 24.82
Protein	0.76**(0.64-0.85)<br>-9.64** ± 15.20	0.73**(0.59-0.82)<br>-4.86* ± 18.89	-	0.80**(0.69-0.87)<br>-14.55** ± 14.59	0.81**(0.71-0.88)<br>-17.51** ± 14.30	0.84**(0.75-0.9)<br>-9.20** ± 17.09	0.73**(0.59-0.82)<br>-0.93 ± 22.80
Carbohydrate	0.71**(0.58-0.82)<br>-11.47** ± 15.50	-	0.78**(0.67-0.86)<br>-10.03** ± 12.60	0.70**(0.55-0.8)<br>-15.76** ± 18.42	0.73**(0.59-0.82)<br>-16.78** ± 16.25	0.80**(0.69-0.87)<br>-4.51* ± 13.47	0.78**(0.66-0.86)<br>-2.58 ± 21.81
**Diet Types**							
Pure Liquid (8)	0.99**(0.93-1.0)<br>-0.38 ± 7.32	1.0**(0.99-1.0)<br>2.88 ± 4.52	0.79*(0.18-0.96)<br>-9.53 ± 17.28	0.79*(0.19-0.96)<br>-0.66 ± 17.65	0.57(-0.23-0.91)<br>-12.82 ± 21.72	0.23(-0.57-0.8)<br>-27.99** ± 30.20	1.00**(0.97-1.0)<br>11.69 ± 32.54
Convenient Diet (30)	0.81**(0.63-0.9)<br>8.30** ± 12.81	-.85**(0.71-0.93)<br>4.18** ± 9.94	0.94**(0.88-0.97<br>1.85 ± 10.41	0.59**(0.29-0.78)<br>-23.65** ± 20.16	0.78**(0.58-0.89)<br>-19.50**± 22.79	0.96**(0.92-0.98)<br>-5.52* ± 13.23	0.74**(0.51-0.87)<br>18.46** ± 15.91
Canned Food (10)	0.15(-0.53-0.71)<br>-6.83* ± 9.16	.95**(0.79-0.99)<br>5.60** ± 4.15	1.0**(0.98-1.0)<br>3.84** ± 4.35	0. 29(-0.42-0.78)<br>-27.27** ± 17.56	0.04(-0.61-0.65)<br>-29.80** ± 14.44	0.78* (0.31-0.95)<br>-17.74** ± 3.92	0.99**(0.96-1.0)<br>29.33** ± 16.50
High School (10)	0.95**(0.8-0.99)<br>0.55 ± 3.84	0.97**(0.89-0.99)<br>5.50** ± 2.64	0.89**(0.6-0.97)<br>0.42 ± 8.33	0.93**(0.74-0.98)<br>-5.32* ± 5.35	0.89**(0.61-0.98)<br>2.22 ± 8.31	0.77*(0.27-0.94)<br>2.72 ± 8.48	0.85* (0.48-0.96)<br>12.77** ± 6.58
Fast Food (10)	-0.22(-0.75-0.48)<br>-18.61** ± 14.93	0.11(-0.56-0.69)<br>1.43 ± 16.78	-0.03(-0.65-0.61)<br>1.30 ± 15.94	0.21(-0.48-0.74)<br>-38.36** ± 18.54	0.23(-0.46-0.75)<br>-30.94**± 18.11	0.76*(0.25-0.94)<br>-1.55 ± 14.77	-0.39(-0.82-0.32)<br>13.29** ± 17.43
Ethnic Food (71)	0.95**(0.92-0.97)<br>-3.22** ± 9.90	0.93**(0.88-0.95)<br>2.01 ± 13.96	0.96**(0.93-0.97)<br>-7.40** ± 8.35	0.92**(0.88-0.95)<br>-9.14** ± 8.53	0.91**(0.86-0.94)<br>-11.73** ± 11.65	0.96**(0.93-0.97)<br>-1.05 ± 7.86	0.97**(0.96-0.98)<br>5.39** ± 12.12
Western Diet (38)	0.85**(0.72-0.92)<br>-1.90 ± 9.66	0.77**(0.6-0.87)<br>2.86*± 14.24	0.86**(0.74-0.92)<br>-5.15** ± 7.57	0.84**(0.72-0.92)<br>-6.53 **± 9.03	0.83**(0.69-0.91)<br>-6.10** ± 11.27	0.84**(0.71-0.91)<br>-0.59 ± 8.10	0.89**(0.8-0.94)<br>5.37**± 13.40
Mexican (10)	0.66*(0.06-0.91)<br>3.40 ± 11.83	0.62(-0.01-0.9)<br>13.04* ± 17.65	0.91**(0.66-0.98)<br>-5.01** ± 3.45	0.83*(0.42-0.96)<br>-6.59 ± 11.83	0.78* (0.29-0.94)<br>-6.45 ± 11.52	0.49(-0.2-0.86)<br>-3.49** ± 3.26	0.83*(0.43-0.96)<br>15.54** ± 8.92
Italian (10)	0.56(-0.11-0.88)<br>-1.31 ± 3.10	0.94**(0.77-0.99)<br>4.46** ± 1.98	-0.17(-0.72-0.51)<br>-2.95 ± 7.29	0.22(-0.47-0.75)<br>-8.11** ± 3.76	0.01(-0.62-0.63)<br>-8.65** ± 5.05	-0.08(-0.68-0.58)<br>-3.51 ± 6.14	0.96**(0.83-0.99)<br>7.89** ± 4.59
Mediterranean (9)	0.03(-0.65-0.68)<br>-6.80 ± 8.68	0.74*(0.14-0.94)<br>-3.11 ± 8.65	0.54(-0.19-0.89)<br>-10.10 ± 9.10	0.06(-0.63-0.7)<br>-8.94 ± 10.88	-0.05(-0.69-0.63)<br>-4.56 ± 18.09	0.62(-0.08-0.91)<br>-3.11 ± 9.58	0.92**(0.66-0.98)<br>1.21 ± 7.62
American (9)	0.46(-0.29-0.86)<br>-3.53 ± 11.07	0.65(-0.02-0.92)<br>-4.27 ± 16.67	0.41(-0.35-0.85)<br>-2.81 ± 8.34	0.21(-0.53-0.77)<br>-2.28 ± 7.41	0.05(-0.64-0.69)<br>-4.41 ± 8.22	0.66(-0.00-0.92)<br>8.39** ± 6.19	0.90*(0.58-0.98)<br>-4.59 ± 19.76
Eastern Diet (33)	0.98**(0.96-0.99)<br>-4.75** ± 10.10	.97**(0.95-0.99)<br>1.03 ± 13.78	0.98**(0.96-0.99)<br>-9.99 ± 8.57	0.98**(0.96-0.99)<br>-12.15 ± 6.88	0.98**(0.96-0.99)<br>-18.22 ± 8.30	0.98**(0.96-0.99)<br>-1.59 ± 7.67	0.99**(0.98-1.0)<br>5.41 ± 10.67
Japanese (10)	0.89**(0.58-0.97)<br>-3.13** ± 2.20	0.96**(0.84-0.99)<br>1.92* ± 2.86	0.99**(0.97-1.0)<br>-4.57** ± 1.61	0.66*(0.06-0.91)<br>-8.34** ± 3.76	0.66*(0.05-0.91)<br>-15.11** ± 2.88	1.00**(0.98-1.0)<br>1.89** ± 1.38	1.00**(0.99-1.0)<br>7.58** ± 4.40
Chinese (10)	0.82*(0.40-0.96)<br>-6.85** ± 2.31	0.95**(0.79-0.99)<br>1.83* ± 2.87	0.93**(0.74-0.98)<br>-10.74** ± 1.57	0.78*(0.30-0.95)<br>-16.71** ± 2.09	0.70*(0.13-0.92)<br>-20.09** ± 1.88	0.66*(0.05-0.91)<br>-2.46 ± 10.33	0.95**(0.80-0.99)<br>7.74** ± 3.88
Korean (13)	0.98**(0.93-0.99)<br>-4.38 ± 16.07	0.97**(0.91-0.99)<br>-0.26 ± 22.16	0.98**(0.95-1.0)<br>-13.57** ± 12.38	0.99**(0.96-1.0)<br>-11.57** ± 9.09	0.98**(0.94-0.99)<br>-19.16** ± 12.77	0.99**(0.97-1.0)<br>-3.58* ± 7.76	0.99**(0.97-1.0)<br>1.96 ± 16.02
Smoothie (22)	0.62*(0.27-0.83)<br>-9.13 ± 23.79	0.46*(0.05-0.74)<br>-7.70 ± 27.16	0.65*(0.32-0.84)<br>-9.86* ± 21.40	0.77**(0.52-0.9)<br>-14.12** ± 23.53	0.79**(0.56-0.91)<br>-12.90** ± 21.37	0.77**(0.52-0.9)<br>-7.80 ± 22.04	0.11(-0.33-0.51)<br>-6.89 ± 33.37

Note: Carb: carbohydrate; Sat Fat: saturated fat; *r*: pairwise for intraclass correlation coefficient; CI: confidence interval; %diff: % difference against Nutrition Data System for Research; M: mean; SD: standard deviation; *r*: correlation; *$p < 0.05$; **$p < 0.001$.

Supplementary Table S2. Bias and agreement between internet-based application and Nutrition Data System for Research per domains of caloric ranges, energy nutrients, and various diets for vitamins Bs (*N*=131).

Parameters (n)	**Thiamin, mg**<br>*r* ** (95%CI)<br>%diff M ± SD	**Riboflavin, mg**<br>*r* ** (95%CI)<br>%diff M ± SD	**Niacin, mg**<br>*r* ** (95%CI)<br>%diff M ± SD	**Pyridoxine, mg**<br>*r* ** (95%CI)<br>%diff M ± SD	**Folate, mcg**<br>*r* ** (95%CI)<br>%diff M ± SD	**Cobalamin, mcg**<br>*r* ** (95%CI)<br>%diff M ± SD
**Calories**						
<1000 (63)	0.83**(0.74-0.9)<br>6.68** ± 11.25	0.71**(0.56-0.81)<br>-0.14 ± 11.51	0.84**(0.74-0.9)<br>2.08 ± 13.03	0.94**(0.9-0.96)<br>-2.75** ± 11.43	0.78**(0.66-0.86)<br>12.79**± 18.86	0.72**(0.58-0.82)<br>-13.43**± 18.61
1000-2000 (50)	0.90**(0.83-0.94)<br>10.16** ± 9.41	0.87**(0.78-0.92)<br>3.47** ± 10.18	0.88**(0.80-0.93)<br>1.23 ± 8.57	0.98**(0.97-0.99)<br>-2.91** ± 8.23	0.95**(0.91-0.97)<br>9.54**± 10.94	0.53**(0.3-0.7)<br>-12.27**± 15.28
>2000 (18)	0.59*(0.21-0.82)<br>-4.44 ± 24.81	0.61*(0.23-0.83)<br>-7.77 ± 23.64	0.68**(0.35-0.87)<br>-7.00 ± 25.08	0.54*(0.13-0.79)<br>-14.18 ± 31.15	0.55*(0.14-0.8)<br>-5.35 ± 27.79	0.44(-0.0-0.74)<br>-17.83* ± 26.04
**Energy Nutrients**						
Fat	0.65**(0.48-0.77)<br>2.27 ± 16.95	0.66**(0.5-0.78)<br>-3.02 ± 16.57	0.71**(0.56-0.81)<br>-4.60* ± 14.94	0.62**(0.45-0.75)<br>-8.76** ± 18.74	0.69**(0.54-0.8)<br>4.18 ± 19.94	0.60**(0.41-0.73)<br>-18.66** ± 19.32
Protein	0.77**(0.65-0.85)<br>3.05 ± 16.66	0.80**(0.69-0.87)<br>-6.17* ± 14.70	0.81**(0.71-0.88)<br>-4.64* ± 14.87	0.74**(0.61-0.84)<br>-10.24** ± 17.48	0.77**(0.66-0.86)<br>2.10 ± 17.62	0.77**(0.65-0.86)<br>-22.90** ± 17.58
Carbohydrate	0.74**(0.6-0.83)<br>1.01 ± 15.70	0.78**(0.7-0.86)<br>-5.14* ± 13.32	0.80**(0.69-0.87)<br>-4.87* ± 13.62	0.75**(0.62-0.84)<br>-10.05** ± 17.39	0.76**(0.64-0.85)<br>1.97 ± 17.00	0.73**(0.6-0.83)<br>-16.33** ± 18.73
**Diet Types**						
Pure Liquid (8)	0.95**(0.76-0.99)<br>6.94 ± 12.73	0.47(-0.35-0.88)<br>-11.68 ± 16.58	0.98**(0.91-1.0)<br>14.12 ± 23.59	1.00**(0.99-1.0)<br>5.91 ± 18.77	0.96**(0.77-0.99)<br>20.05**± 38.13	0.38(-0.44-0.86)<br>-22.04** ± 23.39
Convenient Diet (30)	0.93**(0.86-0.97)<br>8.67**± 11.98	0.92**(0.83-0.96)<br>7.67** ± 10.65	0.92**(0.84-0.96)<br>2.06 ± 9.88	0.80**(0.62-0.9)<br>-1.11 ± 10.32	0.80**(0.62-0.9)<br>0.47** ± 15.55	0.79**(0.59-0.89)<br>-8.11** ± 12.91
Canned Food (10)	0.94**(0.76-0.99)<br>4.35 ± 8.21	0.80*(0.33-0.95)<br>7.54** ± 4.12	0.93**(0.72-0.98)<br>0.88 ± 1.71	0.96**(0.82-0.99)<br>2.93* ± 4.32	0.97**(0.89-0.99)<br>30.51** ± 15.86	0.21(-0.48-0.74)<br>-8.96** ± 1.84
High School (10)	0.95**(0.81-0.99)<br>16.87** ± 5.15	0.93**(0.73-0.98)<br>10.83** ± 7.23	0.88**(0.55-0.97)<br>4.19 ± 7.01	0.91**(0.64-0.98)<br>-2.73 ± 4.54	0.93**(0.72-0.98)<br>19.10** ± 6.64	0.70*(0.12-0.92)<br>-12.51* ± 15.18
Fast Food (10)	0.26(-0.44-0.76)<br>4.78 ± 16.0	-0.03(-0.65-0.61)<br>4.63 ± 16.58	-0.32(-0.79-0.39)<br>1.05 ± 15.97	-0.30(-0.78-0.41)<br>-3.54 ± 16.62	0.43(-0.27-0.83)<br>11.81** ± 16.93	0.01(-0.62-0.63)<br>-2.87 ± 15.82
Ethnic Food (71)	0.97**(0.95-0.98)<br>8.68** ± 10.01	0.97**(0.95-0.98)<br>0.85 ± 8.66	0.95**(0.91-0.97)<br>-0.01 ± 9.09	0.98**(0.97-0.99)<br>-5.06** ± 8.13	0.97**(0.96-0.98)<br>7.10** ± 9.35	0.76**(0.64-0.85)<br>-13.62** ± 16.05
Western Diet (38)	0.96**(0.92-0.98)<br>11.36**± 7.06	0.87**(0.76-0.93)<br>2.28 ± 9.92	0.84**(0.72-0.92)<br>2.26 ± 7.59	0.92**(0.84-0.96)<br>-5.21** ± 6.72	0.91**(0.83-0.95)<br>7.78** ± 9.60	0.77**(0.60-0.88)<br>-12.82**± 14.22
Mexican (10)	0.96**(0.84-0.99)<br>15.27** ± 5.64	0.80* (0.35-095)<br>4.38 ± 7.37	0.96**(0.82-0.99)<br>1.45 ± 2.95	0.56(-0.11-0.88)<br>-3.22 ± 5.15	0.77* (0.28-0.94)<br>11.94** ± 9.62	-0.19(-0.73-0.5)<br>-14.35** ± 4.66
Italian (10)	0.96**(0.84-0.99)<br>7.56** ± 1.59	0.51(-0.17-0.86)<br>1.65 ± 4.83	-0.24(-0.76-0.46)<br>6.51* ± 12.01	0.84*(0.45-0.96)<br>-3.17 ± 4.76	0.93**(0.74-0.98)<br>5.02** ± 2.75	0.03(-0.61-0.65)<br>-9.77* ± 10.05
Mediterranean (9)	0.81**(0.32-0.96)<br>12.97** ± 7.19	-0.22(-0.77-0.52)<br>1.83 ± 14.80	0.95**(0.78-0.99)<br>-1.70 ± 2.66	0.82*(0.35-0.96)<br>-7.82* ± 7.28	0.94**(0.74-0.99)<br>8.68** ± 6.48	0.03(-0.65-0.68)<br>-27.93** ± 16.92
American (9)	0.60(-0.11-0.9)<br>9.60* ± 9.83	0.50(-0.24-0.88)<br>1.08 ± 11.87	0.65(-0.02-0.92)<br>2.40 ± 6.82	0.93**(0.68-0.98)<br>-7.08* ± 8.89	0.70*(0.07-0.93)<br>5.32 ± 15.27	0.57(-0.15-0.89)<br>0.58 ± 6.08
Eastern Diet (33)	0.98**(0.95-0.99)<br>5.60** ± 11.97	0.99**(0.99-1.0)<br>-0.80 ± 6.71	).98**(0.96-0.99)<br>-2.62 ± 10.05	0.99**(0.97-0.99)<br>-4.88** ± 9.61	0.99**(0.97-0.99)<br>6.31** ± 9.14	0.78**(0.59-0.89)<br>-14.55** ± 18.11
Japanese (10)	0.96**(0.84-0.99)<br>-0.28 ± 2.17	0.97**(0.87-0.99)<br>0.97 ± 1.59	1.00**(0.98-1.0)<br>0.97** ± 0.87	1.00**(0.99-1.0)<br>-1.76 ± 2.47	0.99**(0.96-1.0)<br>9.44** ± 2.95	1.0**(0.99-1.0)<br>8.90** ± 3.65
Chinese (10)	0.96**(0.82-0.99)<br>13.70** ± 1.81	0.87* (0.54-0.97)<br>-0.38 ± 2.39	).88**(0.56-0.97)<br>-5.82** ± 1.92	0.97**(0.87-0.99)<br>-7.11** ± 2.21	0.99**(0.97-1.0)<br>3.65** ± 0.83	0.97**(0.89-0.99)<br>-20.62** ± 4.73
Korean (13)	0.98**(0.94-0.99)<br>3.89 ± 17.01	0.99**(0.98-1.0)<br>-2.49 ± 10.40	0.98**(0.93-0.99)<br>-2.91 ± 15.71	0.99**(0.96-1.0)<br>-5.57 ± 15.0	0.99**(0.96-1.0)<br>5.94* ± 14.20	0.95**(0.85-0.99)<br>-27.91** ± 12.88
Smoothie (22)	0.55*(0.17-0.79)<br>-3.77 ± 22.21	0.57*(0.20-0.8)<br>-7.81 ± 21.57	0.66**(0.34-0.85)<br>-4.86 ± 23.20	0.36(-0.07-0.68)<br>-10.38 ± 29.07	0.28(-0.16-0.63)<br>-4.18 ± 25.22	0.58*(0.20-0.8)<br>-17.89** ± 27.84

Note: *r*: pairwise for intraclass correlation coefficient; CI: confidence interval; %diff: % difference against Nutrition Data System for Research; M: mean; SD: standard deviation; *r*: correlation; **p* <0.05; ***p* <0.001.

Supplementary Table S3. Bias and agreement between internet-based application and Nutrition Data System for Research per domains of caloric ranges, energy nutrients, and various diets for methyl donors and vitamins A, C, and D (*N*=131).

Parameters (n)	Methionine^, g	Choline, mg	Glycine, g	Vitamin A, IU	Vitamin C, mcg	Vitamin D, mcg
	r ** (95%CI)	*r* ** (95%CI)	*r* ** (95%CI)	*r* ** (95%CI)	*r* ** (95%CI)	*r* ** (95%CI)
	%diff M ± SD	%diff M ± SD	%diff M ± SD	%diff M ± SD	%diff M ± SD	%diff M ± SD
**Calories**						
<1000 (63)	0.83**(0.74-0.9)	0.88**(0.82-0.93)	0.76**(0.64-0.85)	0.95**(0.92-0.97)	0.85**(0.77-0.91)	0.91**(0.86-0.95)
	-9.97** ± 11.85	-5.30** ± 9.93	-11.43** ± 14.41	34.51** ± 18.60	4.70* ± 25.54	-0.81 ± 12.28
1000-2000(50)	0.90**(0.82-0.94)	0.94**(0.90-0.97)	0.88**(0.80-0.93)	0.99**(0.98-0.99)	0.99**(0.98-0.99)	0.61**(0.41-0.76)
	-5.84** ± 9.63	-2.95** ± 8.34	-7.83** ± 9.84	33.32** ± 19.88	5.13** ± 13.98	2.68** ± 16.35
>2000 (18)	0.67* (0.33-0.86)	0.50*(0.07-0.77)	0.65*(0.29-0.85)	0.50*(0.08-0.77)	0.61*(0.23-0.83)	0.86**(0.68-0.95)
	-12.66** ± 22.51	-6.10 ± 45.37	-14.43** ± 24.44	-9.60 ± 57.99	-13.12 ± 38.08	0.59 ± 16.50
**Energy Nutrients**						
Fat	0.72**(0.58-0.82)	0.48**(0.27-0.65)	0.71**(0.57-0.81)	0.69**(0.54-0.8)	0.69**(0.54-0.8)	0.84**(0.75-0.9)
	-12.49** ± 14.80	-7.23* ± 24.94	-15.35** ± 15.48	22.84** ± 38.59	-0.11 ± 31.11	-1.70 ± 16.26
Protein	0.85**(0.76-0.9)	0.77**(0.66-0.86)	0.84**(0.75-0.9)	0.73**(0.59-0.82)	0.74**(0.61-0.84)	0.86**(0.78-0.91)
	-16.67** ± 11.63	-12.07** ± 13.77	-19.87** ± 12.36	18.49** ± 37.16	-4.82 ± 28.92	-2.28 ± 15.82
Carbohydrate	0.80**(0.69-0.87)	0.74**(0.61-0.83)	0.79**(0.68-0.87)	0.76**(0.64-0.85)	0.81**(0.71-0.88)	0.80**(0.69-0.87)
	-11.83** ± 12.21	-9.94** ± 13.97	-14.68** ± 13.84	21.10** ± 38.60	-1.84 ± 29.26	-0.62 ± 14.36
**Diet Types**						
Pure Liquid (8)	0.64(-0.12-0.93)	0.91*(0.56-0.98)	0.77*(0.13-0.96)	0.98**(0.89-1.0)	0.99**(0.95-1.0)	0.29(-0.52-0.83)
	-18.58* ± 18.21	-7.0 ± 15.99	-16.0 ± 23.29	35.27** ± 30.34	-11.32 ± 26.18	-15.48* ± 22.57
Convenient Diet (30)	0.94**(0.88-0.97)	0.92**(0.84-0.96)	0.92**(0.83-0.96)	0.75**(0.54-0.88)	0.90**(0.81-0.95)	0.83**(0.67-0.92)
	-1.54** ± 10.27	0.66 ± 10.35	-2.51* ± 10.75	46.93** ±16.17	15.17** ± 18.46	4.51 ± 13.94
Canned Food (10)	0.99**(0.98-1.0)	0.98**(0.90-0.99)	0.99**(0.98-1.0)	0.92**(0.71-0.98)	-0.31(-0.78-0.4)	0.65*(0.03-0.91)
	-2.08** ± 1.0	5.06** ± 6.19	0.87 ± 4.62	34.18** ± 14.47	22.51* ± 18.05	-6.82** ± 2.33
High School (10)	0.86*(0.50-0.97)	0.87*(0.54-0.97)	0.80*(0.33-0.95)	0.13(-0.55-0.7)	0.94**(0.75-0.99)	0.56(-0.10-0.88)
	-2.35 ± 9.10	-1.86 ± 5.55	-4.41 ± 10.58	53.82** ± 13.05	-0.44 ± 5.38	12.98** ± 9.18
Fast Food (10)	0.12(-0.55-0.7)	0.37(-0.34-0.81)	0.05(-0.60-0.66)	0.16(-0.52-0.72)	0.99**(0.95-1.0)	0.24(-0.46-0.76)
	-0.20 ± 15.90	-1.22 ± 15.60	-4.0 ± 14.83	52.78** ± 13.83	23.43** ± 18.36	7.38 ± 17.45
Ethnic Food (71)	0.94**(0.91-0.96)	0.97**(0.95-0.98)	0.92**(0.87-0.95)	0.99**(0.98-0.99)	0.92**(0.87-0.95)	0.97**(0.95-0.98)
	-10.17** ± 8.83	-6.3 ** ± 7.05	-12.80** ± 10.54	31.40 ± 16.48	1.85** ± 20.45	2.62** ± 8.34
Western Diet (38)	0.82**(0.67-0.9)	0.86**(0.75-0.93)	0.79**(0.62-0.88)	0.94**(0.89-0.97)	0.89**(0.80-0.94)	0.91**(0.83-0.95)
	-8.21 ± 8.60	-4.88 ± 7.15	-10.11 ± 9.71	38.82** ± 13.55	-3.51 ± 17.93	3.06 ± 9.74
Mexican (10)	0.94**(0.77-0.99)	0.69*(0.10-0.92)	0.90**(0.61-0.98)	0.94**(0.77-0.99)	0.87**(0.53-0.97)	0.18(-0.51-0.73)
	-10.65** ± 1.99	-3.36 ± 5.45	-9.79** ± 2.91	36.40** ± 6.97	-6.04 ± 9.31	3.30 ± 10.72
Italian (10)	-0.23(-0.75-0.47)	-0.06(-0.66-0.59)	-0.34(-0.80-0.37)	0.99**(0.97-1.0)	0.99**(0.97-1.0)	0.29(-0.41-0.78)
	-5.36 ± 8.07	-5.56* ± 6.54	-5.51 ± 9.85	38.05** ± 9.74	1.30 ± 3.89	-2.64 ± 10.43
Mediterranean (9)	0.63(-0.05-0.91)	0.17(-0.55-0.75)	0.85*(0.43-0.97)	0.99**(0.97-1.0)	1.00**(0.99-1.0)	0.66(-0.01-0.92)
	-13.16* ± 11.20	-7.70 ± 8.66	-21.13** ± 6.59	41.53** ± 16.18	-10.94** ± 5.80	9.41** ± 1.93
American (9)	0.51(-0.23-0.88)	0.61(-0.09-0.91)	0.58(-0.14-0.9)	0.86*(0.46-0.97)	0.75*(0.18-0.94)	0.44(-0.32-0.85)
	-3.71 ± 8.42	-2.98 ± 7.95	-4.55 ± 8.70	39.64** ± 20.21	1.38 ± 34.82	2.75 ± 9.88
Eastern Diet (33)	0.97**(0.95-0.99)	0.99**(0.97-0.99)	0.96**(0.91-0.98)	0.99**(0.98-1.0)	0.92**(0.85-0.96)	0.99**(0.99-1.0)
	-12.43** ± 8.67	-7.95** ± 6.67	-15.89** ± 10.75	22.86** ± 15.55	8.03** ± 21.68	2.12** ± 6.46
Japanese (10)	0.99**(0.98-1.0)	0.99**(0.97-1.0)	0.99**(0.98-1.0)	1.00**(0.98-1.0)	1.00**(1.0-1.0)	1.00**(0.99-1.0)
	-7.16** ± 1.94	-6.70** ± 1.36	-6.48** ± 1.65	40.95** ± 12.02	12.37** ± 8.91	1.27** ± 2.27
Chinese (10)	0.97**(0.87-0.99)	0.55(-0.12-0.88)	0.95**(0.80-0.99)	1.00**(1.0-1.0)	1.00**(0.99-1.0)	0.99**(0.96-1.0)
	-13.76** ± 1.52	-5.88 ± 5.37	-19.15** ± 1.58	18.22** ± 5.25	1.44 ± 3.41	3.83** ± 3.58
Korean (13)	0.98**(0.93-0.99)	0.99**(0.97-1.0)	0.97**(0.90-0.99)	0. 99**(0.98-1.0)	0.89**(0.67-0.97)	0.99**(0.97-1.0)
	-15.46** ± 12.71	-10.50** ± 9.15	-20.63** ± 14.05	12.52** ± 10.44	9.77 ± 33.62	1.46 ± 9.71
Smoothie (22)	0.70**(0.40-0.87)	0.41(-0.01-0.71)	0.66**(0.33-0.85)	0.23(-0.21-0.59)	0.54*(0.16-0.79)	0.83**(0.62-0.93)
	-10.49** ± 21.01	-4.88 ± 40.98	-11.79** ± 22.86	-11.46 ± 47.91	-8.18 ± 35.77	-4.73 ± 21.74

Note: ^Methionine RDI derived from a standard weight of 65 kg; *r*: pairwise for intraclass correlation coefficient; CI: confidence interval; %diff: % difference against Nutrition Data System for Research; M: mean; SD: standard deviation; *r*: correlation; **p* <0.05; ***p* <0.001.

Supplementary Table S4. Bias and agreement between internet-based application and Nutrition Data System for Research per domains of caloric ranges, energy nutrients, and various diets for vitamin E and minerals (*N*=131).

**Parameters (n)**	**Vitamin E, mcg**<br>*r* ** (95%CI)<br>%diff M ± SD	**Zinc, mg**<br>*r* ** (95%CI)<br>%diff M± SD	**Calcium, mg**<br>*r* ** (95%CI)<br>%diff M± SD	**Magnesium, mg**<br>*r* ** (95%CI)<br>%diff M ± SD	**Iron, mg**<br>*r* ** (95%CI)<br>%diff M ± SD	**Sodium, mg**<br>*r* ** (95%CI)<br>%diff M ± SD
**Caloric Ranges**						
<1000 (63)	0.81**(0.70-0.88)	0.50**(0.29-0.66)	-0.11(-0.34-0.14)	0.83**(0.73-0.89)	0.72**(0.58-0.82)	0.83**(0.74-0.89)
	7.55** ± 22.56	-12.21** ± 16.74	10.35 ± 41.41	2.22** ± 13.05	6.54** ± 21.09	-18.09** ± 16.56
1000-2000 (50)	0.63**(0.43-0.77)	0.57**(0.35-0.73)	0.81**(0.69-0.89)	0.98**(0.96-0.99)	0.87**(0.78-0.92)	0.50**(0.26-0.68)
	-5.53 ± 22.04	-11.98** ± 10.61	-6.32** ± 11.60	4.02** ± 9.26	3.42** ± 9.53	-15.84** ± 17.39
>2000 (18)	0.53*(0.12-0.79)	0.64*(0.28-0.84)	0.53*(0.12-0.79)	0.55*(0.14-0.80)	0.66*(0.31-0.85)	0.59*(0.19-0.82)
	-11.82 ± 28.51	-14.89* ± 23.66	-8.04 ± 23.10	-8.78 ± 29.71	-9.77 ± 22.78	-36.59** ± 22.07
**Energy Nutrients**						
Fat	0.64**(0.47-0.76)	0.60**(0.42-0.74)	0.26* (0.02-0.47)	0.60**(0.42-0.74)	0.68**(0.53-0.79)	0.51**(0.30-0.67)
	-11.78** ± 25.18	-17.36** ± 17.82	-4.31 ± 29.77	-2.20 ± 19.80	-1.09 ± 18.61	-22.24** ± 17.12
Proteins	0.73**(0.60-0.83)	0.81**(0.71-0.88)	0.25*(0.01-0.47)	0.74**(0.61-0.83)	0.82**(0.73-0.89)	0.72**(0.58-0.82)
	-0.53 ± 21.28	-21.96** ± 13.68	1.06 ± 41.65	-5.60* ± 17.18	-5.09* ± 15.91	-24.65** ± 16.64
Carbohydrate	0.68**(0.52-0.79)	0.75**(0.62-0.84)	0.28*(0.04-0.48)	0.75**(0.62-0.84)	0.81**(0.70-0.88)	0.69**(0.54-0.8)
	-5.29 ± 25.05	-17.42** ± 14.91	-1.53 ± 34.90	-4.62* ± 17.33	-4.47* ± 15.87	-25.93** ± 17.06
**Diet Type**						
Pure Liquid (8)	0.99**(0.93-1.0)	0.49(-0.33-0.89)	-0.32(-0.84-0.49)	0.96**(0.77-0.99)	0.77*(0.14-0.96)	0.84*(0.32-0.97)
	21.70 ± 44.47	-12.66 ± 23.51	-12.76 ± 21.24	3.21 ± 19.25	18.50 ± 46.19	-13.88 ± 43.08
Convenient Diet (30)	0.40*(0.05-0.67)	0.83**(0.68-0.92)	0.93**(0.87-0.97)	0.84**(0.69-0.92)	0.86**(0.72-0.93)	0.93**(0.86-0.97)
	19.65** ± 24.43	-3.99* ± 13.02	-1.47 ± 11.27	10.45** ± 11.41	14.04** ± 12.83	-8.88** ± 13.38
Canned Food (10)	0.51(-0.17-0.86)	0.99**(0.96-1.0)	0.90**(0.61-0.98)	0.99**(0.96-1.0)	0.99**(0.97-1.0)	0.98**(0.9-1.0)
	20.0 ± 21.14	8.50** ± 3.19	0.31 ± 3.09	15.67** ± 5.52	25.71** ± 2.20	-20.02** ± 6.81
High School (10)	0.92**(0.68-0.98	0.90**(0.63-0.98)	0.94**(0.77-0.99)	0.91**(0.66-0.98)	0.92**(0.70-0.98)	0.89**(0.61-0.98)
	2.18 ± 5.56	-10.13** ± 7.42	3.19 ± 7.30	10.22** ± 5.68	9.90** ± 5.45	1.42 ± 7.57
Fast Food (10)	0.27(-0.43-0.77)	-0.25(-0.76-0.45)	0.20(-0.49-0.74)	0.06(0.59-0.67)	-0.16(-0.72-0.52)	0.25(-0.45-0.76)
	41.13** ± 20.09	-10.33 ± 14.86	-7.90 ± 16.52	5.45 ± 17.29	6.52 ± 16.21	-8.05 ± 14.71
Ethnic Food (71)	0.93**(0.89-0.96)	0.91**(0.86-0.94	0.22(0.01-0.43)	0.98**(0.97-0.99)	0.97**(0.94-0.98)	0.91**(0.86-0.95)
	-8.58** ± 10.84	-16.45** ± 11.44	7.99 ± 39.24	-0.33 ± 9.25	0.58 ± 9.95	-18.38** ± 10.23
Western Diet (38)	0.64**(0.4-0.8)	0.78**(0.61-0.88)	0.60**(0.34-0.77)	0.80**(0.64-0.89)	0.96**(0.92-0.98)	0.91**(0.84-0.95)
	7.91** ± 12.15	-16.28** ± 10.44	-5.63* ± 15.05	0.01 ± 9.12	1.80 ± 8.51	-12.18** ± 7.86
Mexican (10)	-0.25(-0.76-0.45)	0.76*(0.26-0.94)	0.74*(0.21-0.93)	0.89**(0.59-0.97)	0.96**(0.82-0.99)	0.88**(0.55-0.97)
	4.04 ± 18.06	-15.31** ± 5.61	-11.41** ± 10.40	2.23 ± 5.64	5.36** ± 3.82	-8.01** ± 5.55
Italian (10)	0.99**(0.94-1.0)	-0.42(-0.83-0.29)	0.44(-0.26-0.84)	0.74*(0.20-0.93)	0.40(-0.31-0.82)	0.62(-0.01-0.9)
	11.91** ± 3.61	-12.10** ± 11.32	-7.64** ± 6.25	0.46 ± 4.09	6.76** ± 4.97	-16.40** ± 3.35
Mediterranean (9)	0.95**(0.78-0.99	-0.25(-0.79-0.49)	-0.25(-0.78-0.5)	0.61(-0.09-0.91)	0.75*(0.18-0.94)	-0.26(-0.79-0.49)
	0.74 ± 6.09	-23.17** ± 13.26	1.89 ± 21.36	2.12 ± 8.16	-8.90** ± 5.36	-12.23* ± 10.70
American (9)	0.81*(0.30-0.96)	0.22(-0.52-0.77)	0.12(-0.59-0.73)	0.59(-0.13-0.9)	0.47(-0.28-0.86)	0.17(-0.55-0.75)
	14.93** ± 10.60	-15.11** ± 8.41	-4.51 ± 17.70	-5.07 ± 14.93	3.03 ± 9.22	-12.06** ± 8.94
Eastern Diet (33)	0.98**(0.96-0.99	0.95**(0.89-0.97)	0.34(0.01-0.61)	0.99**(0.98-1.0)	0.98**(0.95-0.99)	0.97**(0.95-0.99)
	9.36** ± 9.22	-16.65** ± 12.67	23.67 ± 51.30	-0.72 ± 9.53	-0.82 ± 11.36	-25.53** ± 7.68
Japanese (10)	0.93**(0.72-0.98	0.98**(0.93-1.0)	0.99**(0.96-1.0)	1.0**(0.99-1.0)	0.93**(0.71-0.98)	0.65*(0.03-0.91)
	15.91** ± 6.21	-5.54** ± 1.16	2.44 ± 0.79	3.76** ± 0.93	6.93** ± 4.03	-27.38** ± 3.71
Chinese (10)	0.98**(0.9-0.99)	0.66*(0.05-0.91)	0.97**(0.86-0.99)	0.86*(0.49-0.97)	0.93**(0.71-0.98)	0.79*(0.31-0.95)
	1.35 ± 3.21	-22.09** ± 1.80	-8.44** ± 1.51	-0.98 ± 3.19	-3.74** ± 1.71	-21.70** ± 3.30
Korean (13)	0.99**(0.98-1.0)	0.96**(0.87-0.99)	0.22(-0.38-0.69)	0.99**(0.97-1.0)	0.98**(0.94-0.99)	0.98**(0.94-1.0)
	10.49** ± 9.98	-21.01** ± 16.62	64.70* ± 62.91	-3.98 ± 14.34	-4.54 ± 16.05	-27.05** ± 11.01
Smoothie (22)	0.44*(0.02-0.73)	0.62*(0.27-0.82)	0.57*(0.2-0.8)	0.24(-0.21-0.6)	0.61*(0.25-0.82)	0.67**(0.35-0.85)
	9.39 ± 26.64	-11.24 ± 23.16	-10.46 ± 20.70	-6.03 ± 27.38	-9.25* ± 20.28	-41.27** ± 17.60

Note: *r*: pairwise for intraclass correlation coefficient; CI: confidence interval; %diff: % difference against Nutrition Data System for Research; M: mean; SD: standard deviation; *r*: correlation; **p* <0.05; ***p* <0.001.

Supplementary Table S5. Progression on selecting significant factors contributing to the differences between Internet-based Application and Nutrition Data System for Research on total calories.

Parameters	Logistic Regression original model				Generalized Regression Elastic Net model validation			
	p (χ^2)	MR	AICc	AUC	p (χ^2)	MR	AICc	AUC
**Caloric Range, % Difference**								
< 1000	0.5467	0.5556	44.75	0.6335	0.5657	0.5556	44.75	0.6335
> 2000	0.1798				0.1875			
**Energy Nutrients, % Difference**								
Fat	0.0001	0.2593	36.63	0.8636	<0.0001	0.2593	35.83	0.8636
Carbohydrate	0.0003				<0.0001			
Protein	0.0454				0.0489			
**Diet Types**								
Fast Food	0.9148	0.4074	64.03	0.5142	0.0262	0.4074	44.71	0.5142
Chinese	0.9148				<0.0001			
**Combined Factor Model: 3 Factors** (Table 3)								
< 1000 caloric range	0.0318	0.1852	30.29	0.8920	0.0313	0.1852	30.29	0.8920
Protein % Difference	0.0118				0.0109			
Fat % Difference	<0.0001				<0.0001			
**Combined Factor Model: 4 Factors**								
< 1000 caloric range	0.0213	0.1852	33.09	0.8977	0.0212	0.1852	33.09	0.8977
Protein % Difference	0.0360				0.0332			
Fat % Difference	<0.0001				<0.0001			
Chinese	0.9073				<0.0001			

Note: MR: misclassification rate; AICc: Akaike's information criterion with corrections; AUC: Area under the curve

Supplementary Table S6. Progression on selecting significant factors contributing to the differences between Internet-based Application and Nutrition Data System for Research on folate.

Parameters	Logistic Regression original model				Generalized Regression Elastic Net model validation			
	p (χ^2)	MR	AICc	AUC	p (χ^2)	MR	AICc	AUC
**Caloric Range, % Difference**								
< 1000	0.0038	0.4074	44.53	0.6382	0.0454	0.4074	44.05	0.6382
**Energy Nutrients, % Difference**								
Carbohydrate	0.1436	0.3333	42.66	0.7697	0.1428	0.3333	42.66	0.7697
Protein	0.0529				0.0533			
Fiber	0.2859				0.2810			
**Diet Types**								
Canned food	0.8927	0.4815	45.67	0.8618	<0.0001	0.4815	45.67	0.8618
High School	0.8927				<0.0001			
Italian	0.8926				<0.0001			
Chinese	0.8783				<0.0001			
Smoothie	0.0342				0.0342			
**Combined Factor Model: 3 Factors** (Table 4)								
Calories range < 1000	0.0033	0.2963	38.52	0.9046	0.0033	0.2963	38.52	0.9046
Carbohydrate % Difference	0.0001				0.0001			
Italian diet	0.8842				<0.0001			
**Combined Factor Model: 4 Factors**								
Calories range < 1000	0.0119	0.2963	38.88	0.9112	0.0119	0.2963	38.88	0.9112
Carbohydrate % Difference	0.0004				0.0003			
Italian diet	0.9085				<0.0001			
Chinese diet	0.9039				<0.0001			

Note: MR: misclassification rate; AICc: Akaike's information criterion with corrections; AUC: Area under the curve

Supplementary Table S7. Progression on selecting significant factors contributing to the differences between Internet-based Application and Nutrition Data System for Research on cobalamin.

Parameters	Logistic Regression original model				Generalized Regression Elastic Net model validation			
	p (χ^2)	MR	AICc	AUC	p (χ^2)	MR	AICc	AUC
**Caloric Range, % Difference**								
> 2000	0.1480	0.5185	42.21	0.5167	0.6025	0.5185	41.84	0.5167
**Energy Nutrients, % Difference**								
Protein	<0.0001	0.2593	36.33	0.7583	<0.0001	0.2593	35.86	0.7583
**Diet Types**								
Mexican	0.0269	0.2963	45.45	0.8000	0.0269	0.2963	45.45	0.8000
Mediterranean	0.0269				0.0269			
American	0.8968				<0.0001			
Japanese	0.8830				<0.0001			
Chinese	0.8716				<0.0001			
Korea	0.0134				0.0134			
**Combined Factor Model: 3 Factors** (Table 5)								
Protein % Difference	<0.0001	0.2593	37.73	0.8083	<0.0001	0.2593	37.42	0.8083
American	0.9124				0.0002			
Japanese	0.8929				<0.0001			
**Combined Factor Model: 4 Factors**								
Protein % Difference	<0.0001	0.2593	40.58	0.8111	<0.0001	0.2593	40.42	0.8111
American	0.8910				<0.0001			
Japanese	0.8671				<0.0001			
Chinese	0.9105				<0.0001			
**Combined Factor Model: 5 Factors**								
Protein % Difference	<0.0001	0.2222	42.87	0.8361	<0.0001	0.222	42.56	0.8361
Mexican	0.0290				0.0363			
American diet	0.8915				0.0002			
Japanese diet	0.8680				<0.0001			
Chinese diet	0.9093				0.0111			

Note: MR: misclassification rate; AICc: Akaike's information criterion with corrections; AUC: Area under the curve

Supplementary Table S8. Summary of significant factors contributing to the differences between Internet-based Application and Nutrition Data System for Research on major nutrients.

Parameters	Carbohydrate	Protein	Fat	Saturated Fat	Cholesterol	Fiber
	% Difference	% Difference	% Difference	% Difference	% Difference	% Difference
**Caloric range**						
1000-2000	-	0.0086	-	-	-	0.0219
**Energy Nutrients, % Difference**						
Total Calories	<0.0001	<0.0001	-	0.0027	-	-
Carbohydrate	n/a	-	-	-	-	<0.0001
Protein	-	n/a	-	-	0.0053	-
Fat	-	-	n/a	0.0002	0.0018	-
Saturated fat	-	-	<0.0001	n/a	-	-
Cholesterol	-	0.0114	0.0020	-	n/a	-
Fiber	<0.0001	-	-	-	-	n/a
**Diet Types**						
Pure liquid	-	-	-	-	-	-
Canned-food	-	<0.0001	-	-	<0.0001	<0.0001
Fast foods	-	-	0.0273	-	-	-
Italian	<0.0001	-	-	-	-	-
American	-	-	-	-	<0.0001	-
Japanese	-	-	-	-	<0.0001	-
Korean	-	-	-	0.0042	-	-
MR	0.2222	0.1111	0.1111	0.1111	0.2222	0.1481
AICc	32.6010	32.2818	27.34	23.9683	36.8539	30.5846
AUC	0.8097	0.9000	0.9278	0.9753	0.9176	0.9056

Note: MR: misclassification rate; AICc: Akaike's information criterion with corrections; AUC: Area under the curve. Insignificant parameters are excluded: caloric ranges of <1000 and >2000, pure liquid diet, high school diet, Mexican diet, Mediterranean diet, Chinese diet, and smoothie-added diet.

Supplementary Table S9. Summary of significant factors contributing to the differences between Internet-based Application and Nutrition Data System for Research on methyl-donors and co-factors.

Parameters	Thiamin<br>% Difference	Riboflavin<br>% Difference	Niacin<br>% Difference	Pyridoxine<br>% Difference	Choline<br>% Difference	Glycine<br>% Difference	Zinc<br>% Difference
**Energy Nutrients, % Difference**							
Total Calories	0.0018	-	<0.0001	<0.0001	<0.0001	-	0.0148
Carbohydrate	-	<0.0001	-	-	-	-	-
Protein	-	0.0015	-	-	-	<0.0001	0.0002
Fat	-	-	-	-	-	-	-
Fiber	0.0057	-	-	-	-	-	-
**Diet Types**							
Canned-food	-	-	-	<0.0001	<0.0001	<0.0001	<0.0001
High School	<0.0001	<0.0001	-	-	-	-	-
Japanese	-	-	-	-	-	<0.0001	<0.0001
Chinese	<0.0001	-	<0.0001	-	-	-	-
MR	0.2222	0.1852	0.1111	0.0741	0.1111	0.0370	0.1852
AICc	35.7070	29.7176	27.0972	24.2056	25.5887	19.7628	34.5265
AUC	0.9091	0.9056	0.8971	0.9176	0.8892	0.9659	0.8750

Note. MR: misclassification rate; AICc: Akaike's information criterion with corrections; AUC: Area under the curve. Insignificant parameters are excluded: all calories ranges, pure liquid diet, fast food diet, Mexican diet, Italian diet, Mediterranean diet, American diet, Korean diet and smoothie-added diet.

Supplementary Table S10. Summary of significant factors contributing to the differences between Internet-based Application and Nutrition Data System for Research on other vitamins and minerals.

Parameters	Vitamin A	Vitamin D	Vitamin E	Vitamin C	Calcium	Magnesium	Iron	Sodium
	% Difference	% Difference	% Difference	% Difference	% Difference	% Difference	% Difference	% Difference
**Energy Nutrients, % Difference**								
Total calories	-	-	-	-	-	0.0012	-	-
Protein	0.0039	-	-	-	-	0.0101	0.0050	-
Fat	-	-	0.0032	-	-	-	-	-
Saturated Fat	-	-	-	-	-	-	-	0.0214
Cholesterol	-	0.0002	-	-	<0.0001	-	-	-
Fiber	-	-	-	0.0042	-	0.0325	-	-
**Diet Types**								
Canned-food	-	<0.0001	-	-	-	<0.0001	-	-
High School	-	-	-	-	-	<0.0001	<0.0001	<0.0001
Fast-food	-	-	<0.0001	<0.0001	-	-	-	
Mexican	-	-	-	0.0247-	0.0154	-	-	<0.0001
Italian	-	-	<0.0001	-	-	-	-	-
Mediterranean	-	<0.0001	-	-	-	-	<0.0001	-
Chinese	-	-	-	-	<0.0001	-	-	-
Korean	<0.0001	-	-	-	-	-	-	-
Smoothie-added	<0.0001	-	-	-	-	-	-	-
MR	0.1852	0.1852	0.2222	0.2222	0.3333	0.2222	0.1111	0.2963
AICc	31.1746	35.9705	37.1307	38.3823	41.7396	32.8717	33.4985	39.1133
AUC	0.8516	0.8472	0.8040	0.8022	0.7250	0.8892	0.8977	0.7500

Note. MR: misclassification rate; AICc: Akaike's information criterion with corrections; AUC: Area under the curve. Insignificant parameters are excluded: all caloric ranges of<1000, 1000 – 2000, and >2000, pure liquid diet, canned food diet, American diet, and Japanese diet.

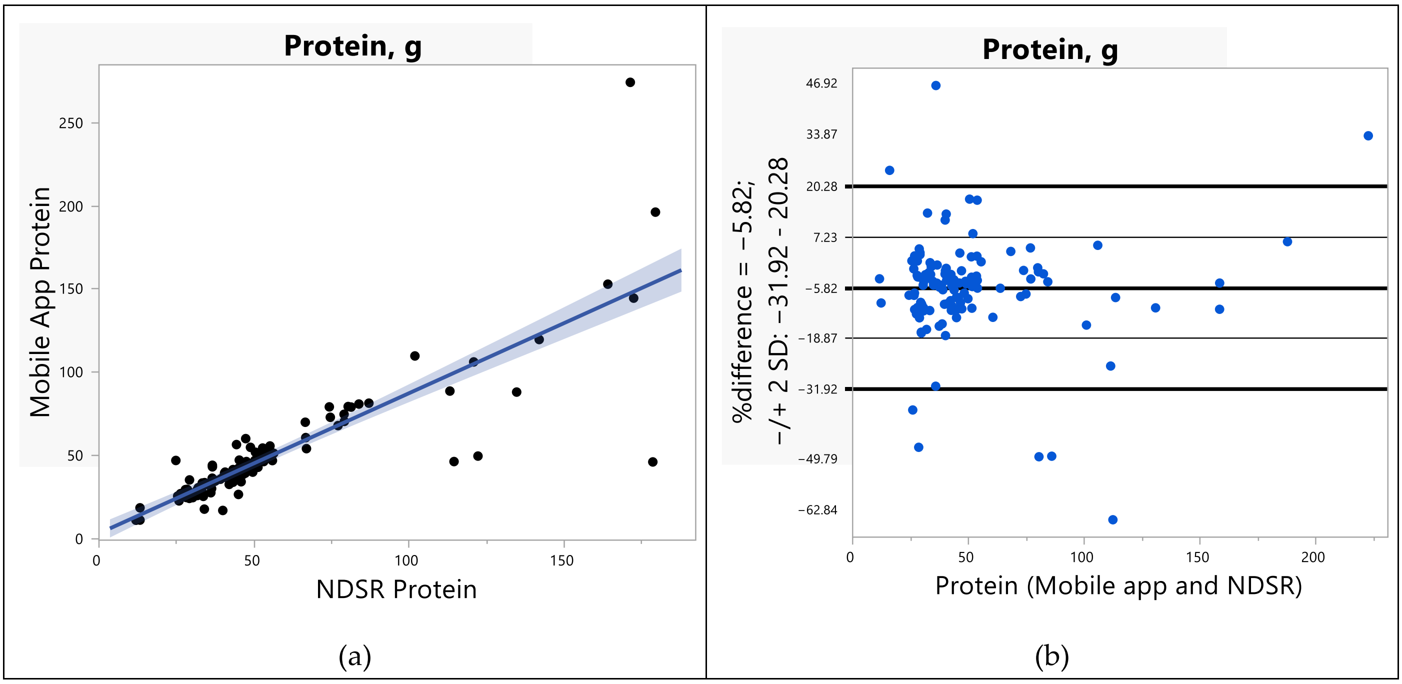

Supplementary Figure S1. (a) Correlation, (b) Bland-Altman plots between internet-based application and Nutrition Data System for Research (NDSR) for protein (a, b).

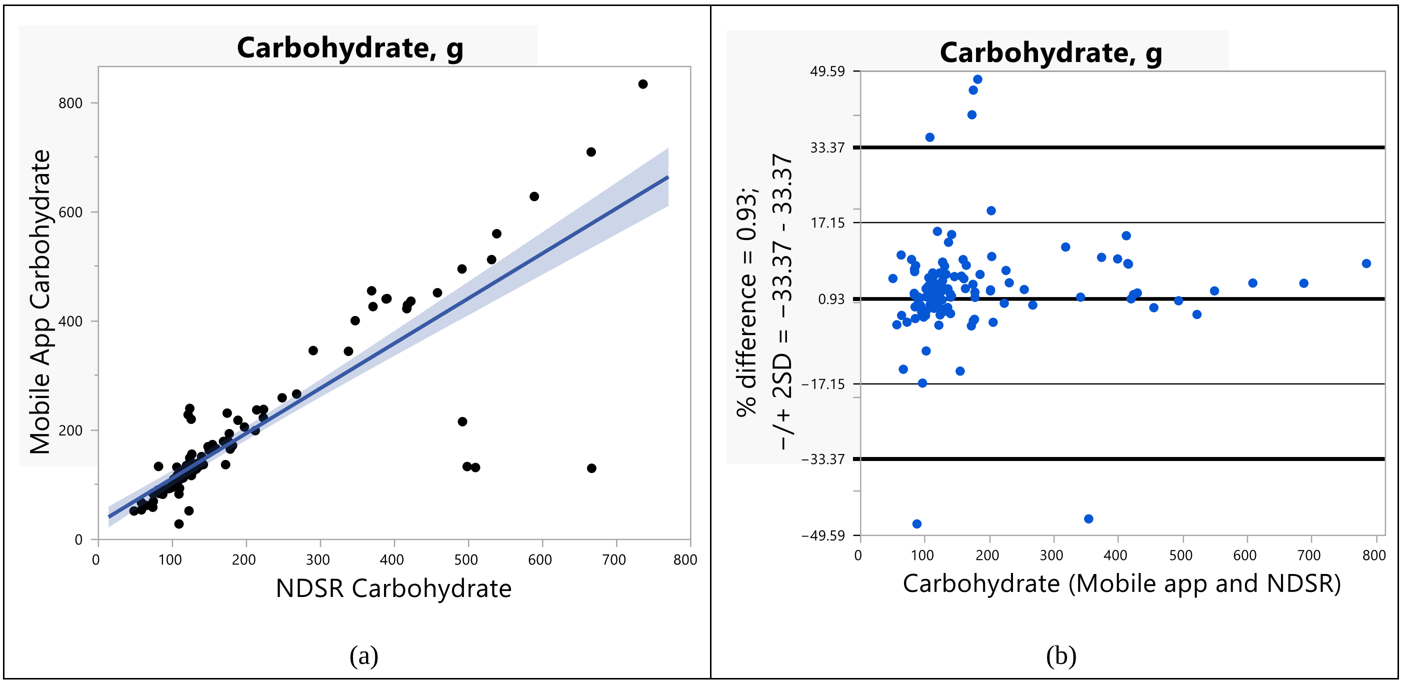

Supplementary Figure S2. (**a**) Correlation, (**b**) Bland-Altman plots between internet-based application and Nutrition Data System for Research (NDSR) for carbohydrate (a, b).
